# Summary and Recommendations: *Highlights of the Asthma Summit 2009: Beyond the Guidelines*

**DOI:** 10.1097/WOX.0b013e3181d046dd

**Published:** 2010-02-15

**Authors:** Michael A Kaliner, Judith R Farrar

**Affiliations:** 1 Institute for Asthma and Allergy, Wheaton and Chevy Chase, MD, and George Washington University School of Medicine, Washington, DC; 2 LifeSciences Press, Canandaigua, NY

## 

Despite intense attention over the past 10-20 years, asthma still offers many potential new targets and approaches for improved treatment. Bronchospasm is the most obvious target, which is amenable to treatment that relaxes airway smooth muscle. Inflammation is the most emphasized aspect of the disease and is used to direct maintenance therapy at the underlying facets of asthma. However, emerging evidence indicates that inflammation itself represents a more complex target than previously understood. The inflammation of asthma is multicellular, and responses to treatment can vary by cell type, so that treatment approaches should be expanded to reflect these differences between individual patients. In addition, no currently available therapies address injury repair, which is also multicellular and contributes to progressive loss of lung function, a component of risk.

It is also apparent that personalized and flexible treatments for patients with asthma are equally important. In fact, many patients are already practicing their own "brand(s)" of personalized and flexible therapy; as indicate by refill rates for short-acting bronchodilators and maintenance medications. It is widely recognized that full compliance with recommendations is only occasionally followed by many, if not most, asthma sufferers. One important outcome of the discussion at the Asthma Summit was that physicians who treat patients with asthma should be willing to work with patients to develop flexible options for treatment that maintain optimal asthma control. This approach requires patient education, regular evaluation of asthma control, is best done in conjunction with home peak flow monitoring, and requires consideration of a range of treatment options that make sense for the individual patient. Specific recommendations from the Asthma Summit toward this goal include the following:

• Individual phenotypes and/or clinical demographic predictors should be taken into consideration when determining appropriate treatment. The goal is to look at specific patterns of symptoms and responses, plus to determine a patient's willingness to stay on regular use of medications to create the most educated treatment plan.

• Asthma control needs to be monitored regularly. Control questionnaires for patients, like the Asthma Control Test (ACT), are helpful, as is the use of home peak flow monitoring. Among the criteria we need to look at now are how frequently patients exacerbate, need urgent care visits, Emergency Department visits or hospitalizations, or use prednisone bursts (as opposed to hospitalizations or urgent care visits). The idea of monitoring symptom control and pulmonary function in tandem to best manage patients is evolving as the best way to follow patients.

• Educating the patient is critical for success. The patient needs to understand how much better their quality of life can be when symptoms are not simply tolerated and no longer interfere with daily activities. Despite 2 decades of emphasizing airway inflammation, patients are still unaware of the chronicity of asthma and the need for ongoing treatment, even if the use of the treatment can be flexible.

• There is room for improvement in recognizing severe disease. Use of SABA and prednisone bursts are both important, and pharmacist involvement may be a good mechanism to identify patients for specialist referral. Pharmacists can also be quite helpful if refill rates are shared with prescribing physicians as one way for compliance to be estimated.

• In terms of specialty treatment and referrals, establishing good working relationships between the pulmonary and the allergy communities is important.

There remains considerable variability in the practice of asthma management, despite multiple reiterations of guidelines for managing asthma; and that variability is associated with poor outcomes and high costs. It must be recognized that although current guidelines provide an excellent reference to the clinician seeking to better understand disease mechanisms and evidence-based approaches to treatment, in practical terms the hundreds of pages in these documents are not particularly useful, especially for the majority of general practice physicians who see only a relatively few asthmatics. Shorter, more focused, guidelines emphasizing updated standards of care are still needed, with information presented from an operational perspective. The experts at the Asthma Summit specifically noted the following as missing from current guidelines:

• How lifestyle change can affect asthma outcomes should be included, with specific examples. This is a topic that is included in other guidelines for managing chronic diseases (eg, cholesterol).

• More information should be given on allergy management including immunotherapy and its role in treating asthma. This is a case where over-caution can be damaging to practice, that is, limiting discussion because of lack of evidence-based documentation in the literature can preclude treatment that has been helpful for many patients. It was agreed that immunotherapy is currently guided by incomplete evidence; however, it was also agreed that allergy assessment followed by appropriate immunotherapy has helped many patients with allergic asthma. Disregarding the potential importance of allergy in the management of asthma can limit success and commit the patient to recurrent disease that could otherwise be improved.

• Step down therapy needs to be better addressed. There are seasonal patterns that drive increased treatment, but the recommendations for step up, step down are not specifically discussed with regard to stepping down. One example is viral infection, particularly the interplay with allergy as a risk factor for having an asthma exacerbation. Both patients and clinicians need to better understand what this means in terms of day-to-day therapy.

In summary, despite multiple reiterations of asthma guidelines, control is still not achieved in the majority of asthma patients, particularly those with more severe disease. On the other hand, flexible dosing might be a much more appropriate method to treat the milder or moderate asthmatic, and impressive results from Europe suggest that this approach should be applied within the United States as well. The time may be now to consider the concept of a global, universal guideline for asthma as a way forward to develop a flexible and personalized approach that will ultimately improve patient care.

